# Urban landscapes tend to increase the presence of pathogenic protozoa, microsporidia and viruses, but likely decrease the abundance of viruses in wild bees and wasps

**DOI:** 10.1111/1744-7917.70137

**Published:** 2025-07-27

**Authors:** Andrea Ferrari, Giovanni Cilia, Carlo Polidori

**Affiliations:** ^1^ Department of Environmental Science and Policy (ESP) University of Milan Milan Italy; ^2^ CREA Research Centre for Agriculture and Environment (CREA‐AA) Bologna Italy

**Keywords:** *Apicystis bombi*, DWV, green fragmentation, *Nosema ceranae*, urban heat island, urbanization

## Abstract

Pathogens are shared between wild bees and wasps but little is known about how urbanization affects their occurrence. Here, the role of temperature and fragmentation of green areas, both associated with urbanization, in modulating pathogen loads was investigated. Twelve pathogens were investigated in the bees *Anthophora plumipes* Pallas, 1772, *Halictus scabiosae* (Rossi, 1790), *Osmia cornuta* (Latreille, 1805), and the wasp *Polistes dominula* (Christ, 1791) sampled across an urbanization gradient in a metropolitan area of northern Italy. Overall, the relative presence/abundance of the pathogens were found to be species specific, as were the responses to urbanization. *Anthophora plumipes* and *O. cornuta* had a higher occurrence probability of the neogregarine protozoan *Apicystis bombi* in more fragmented urban areas. In the same bee species, both temperature and the fragmentation of green areas reduced the number of copies of the deformed wing virus (DWV). In *H. scabiosae* and *P. dominula*, higher temperature increased respectively the likelihood of occurrence of DWV and chronic bee paralysis virus (CBPV). In addition, the viruses were found to be replicative in all samples tested. The results show a consistent presence of pathogens in the four target species, and that urbanization plays a role in modulating the pathogen load. Although transmission pathways could not be considered here, it may be suggested that appropriate management of urban areas may buffer wild insects from potentially harmful pathogens. Whether the presence of such pathogens also results in symptomatic phenotypes remains to be determined in laboratory experiments.

## Graphical abstract

• The bees *Anthophora plumipes* and *Osmia cornuta* had a higher occurrence probability of the neogregarine protozoan *Apicystis bombi* in more fragmented urban areas.• In the bee *Halictus scabiosae* and wasp *Polistes dominula*, hotter urban areas increased the likelihood of occurrence of viruses.• The viruses were found to be replicative in the samples, and the number of copies decreased in more heavily urban areas.

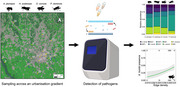

## Introduction

There has been increasing evidence of insect declines (Hallman *et al.*, 2017), with potential negative effects of ecosystem services (Elizalde *et al.*, 2020) provided by flower‐visiting insects such as bees (pollination) or wasps (regulation of arthropod populations, including pests; pollination) (Hanley *et al.*, 2015; Brock *et al.*, 2021; Borchardt *et al.*, 2024). The reasons for this decline are complex and often difficult to disentangle (Yang *et al.*, 2021), but there is evidence that changes in land‐use, such as urbanization, affect bees and wasps from the community to the individual level (Ferrari & Polidori, 2022, 2024, 2025; Geppert *et al.*, 2023; Polidori *et al.*, 2023; Gil‐Tapetado *et al.*, 2024; Ferrari *et al.*, 2024a, 2024b).

The European honeybee (*Apis mellifera* Linnaeus 1758) is the world's most widespread pollinator (Hung *et al.*, 2018). Because of its economic value, its health has been increasingly monitored in the last 20 years, leading to the discovery of numerous pathogens affecting *A. mellifera*, such as viruses, bacteria, fungi or protozoa (Boncristiani *et al.*, 2020). The presence of such pathogens can have several negative effects on honeybees, ranging from morphological changes to the loss of entire colonies (Goblirsch *et al.*, 2013; Desai & Currie, 2016). Examples of the most studied pathogens include deformed wing virus (DWV), which causes irreversible wing deformation (Yue & Genersch, 2005), and the unicellular microsporidium *Nosema ceranae* (Microsporidia: Nosematidae), an intracellular parasite that infects the bee gut and alters digestive functions and the microbiome (Castelli *et al.*, 2020). In addition, bumblebees, which include some of the world's most extensively managed species, are often infected with the protozoan *Apicystis bombi* (Apicomplexa: Neogregarinorida) and *Crithidia bombi* (Kinetoplastida: Trypanosomatidae) (Lipa & Triggiani, 1996; Schmid‐Hempel, 2001; Tiritelli *et al.*, 2025).

The high abundance of honeybees and bumblebees makes them potential carriers of pathogens that they can transmit to wild bees and wasps (Durrer & Schmid‐Hempel, 1994; Graystock *et al.*, 2020; Nanetti *et al.*, 2021a). Evidence suggests that viruses and fungi are more likely to be transmitted to non‐*Apis* pollinators, compared with protozoans, with examples of widespread occurrence of DWV, chronic bee paralysis virus (CBPV), black queen cell virus (BQCV), and *N. ceranae*; and a lower occurrence of protozoans such as *Lotmaria passim* or *C. bombi* (Cilia *et al.*, 2022; Tiritelli *et al.*, 2024). Wild insects can acquire pathogens in different ways. For nectar‐feeders, flowers provide a platform for the spread of pathogens (Figueroa *et al.*, 2019; Burnham *et al.*, 2021). Conversely, predators may become infected during predation (Mazzei *et al.*, 2018; Power *et al.*, 2023; Cilia *et al.*, 2024).

However, little is still known about how urbanization can modulate the occurrence of these pathogens in wild bees and wasps (Goulson *et al.*, 2012; Ivers *et al.*, 2022; Tommasi *et al.*, 2023). Urban habitats present characteristics that may shape pathogen occurrence albeit the exact routes of pathogen transmission often remain to be elucidated (Wilfert *et al.*, 2021). For example, urban landscapes with more fragmented green areas should increase the likelihood of pathogen occurrence by concentrating high densities of insects in small patches of green, known as the “amplification effect” (AE) (Durrer & Schmid‐Hempel, 1994; Becker *et al.*, 2015). On the other hand, higher temperatures promote the expression of immune response genes in bees, suggesting a positive effect of warmer temperatures on immune response (Xu & James, 2012). High temperatures may also reduce the viability of pathogens, like viruses or protozoans, in the environment (Palmer‐Young *et al.*, 2018; Dalmon *et al.*, 2019). Therefore, hotter urban areas (urban heat island effect, UHI) may reduce viral presence and/or abundance.

This study aims to investigate the role of urbanization in modulating the abundance (number of DNA/RNA copies), presence (transformed abundances into 0‒1), and prevalence (number of infected individuals out of the total sampled in a site) of pathogens in three wild bee and one wasp species. Samples were collected across a gradient of green fragmentation and temperature (urbanization) using as a model city Milan (Italy). The aims are (i) to identify the presence of 6 viruses, 4 protozoa and 1 microsporidium and 1 fungus in the target species and (ii) to test the UHI and the AE hypothesis on pathogen abundance, presence, and prevalence. In addition, viral replication in the sampled specimens was checked.

## Materials and methods

### Sampling activity

**Fig. 1:**
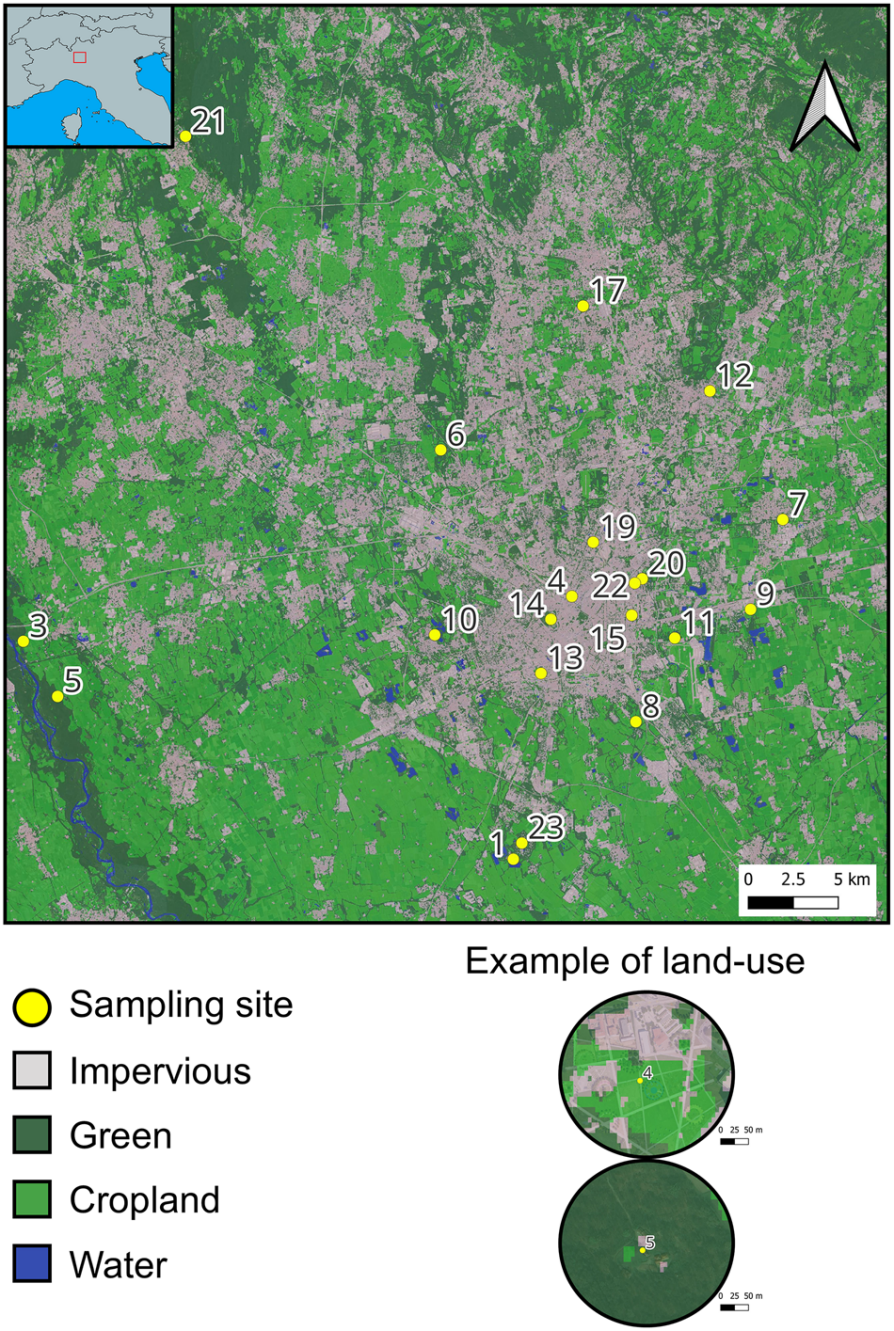
Representative land‐use map showing the geographical location of the sampling sites (indicated by their ID next to the yellow dots) in the metropolitan area of Milan (the central urbanized area in gray). At the top is a schematic representation of the sampling location in northern Italy. On the right are two examples of a highly urbanized site (ID 4) and a less urbanized site (ID 5).

Twenty sampling sites (Fig. 1) were selected across the metropolitan city of Milan situated in Lombardy, northern Italy. Each sampling site was separated by at least 1 Km to ensure spatial independence (i.e., nonoverlapping land‐use buffers to avoid spatial autocorrelation, see next section). Sampling sites were separated on average by 17.61 ± 11.3 Km (mean ± SD), thus ensuring independence between the samples. Sampling took place from March 7 to June 24, 2022. Insects were sampled with an entomological net on flowers or grass, placed in 1.5 mL centrifuge tubes and preserved in >95% ethanol.

Because of their local abundance and ease of identification in the field, four target Hymenoptera species were selected for this study: (i) males of the solitary ground‐nesting bee *Anthophora plumipes* (Apidae) (Pallas, 1772); (ii) female workers of the primitively eusocial, ground‐nesting bee *Halictus scabiosae* (Halictidae) (Rossi, 1790); (iii) females of the solitary cavity‐renter bee *Osmia cornuta* (Megachilidae) (Latreille, 1805); and (iv) female workers of the eusocial aerial‐nesting wasp *Polistes dominula* (Vespidae) (Christ, 1791).

A total of 112 males of *A. plumipes* from 9 sites, 127 females of *H. scabiosae* from 13 sites, 174 females of *O. cornuta* from 8 sites, and 93 females of *P. dominula* from 9 sites were analyzed.

### Landscape characterization

Sampling sites were characterized in terms of land use according to DUSAF6.0 (https://www.dati.lombardia.it/Territorio/Dusaf‐6‐0‐Uso‐del‐suolo‐2018/7rae‐fng6) which contains a land‐use map with a resolution of 20 m. Land‐use variables were extracted from 500 m buffers around the central coordinate of each sampling site. This buffer was chosen because likely describe the environment in which these species forage and nest (Greenleaf *et al.*, 2007, and already used by, e.g., Ferrari *et al.*, 2024a,b). Land use was categorized as impervious (i.e., cemented areas), green (i.e., vegetated areas), and water bodies. The edge density of green areas was calculated as the ratio between the perimeter and the area of the vegetated patches in each buffer: the higher its value, the more fragmented the vegetation is. In addition, the mean site temperature for the sampling activity (March–June 2022) was retrieved from AppEEARS (https://appeears.earthdatacloud.nasa.gov/) through the product MOD11A1.061 “Land Surface Temperature & Emissivity.”

The degree of urbanization was first estimated by the ratio of green to cemented areas (i.e., urban areas had lower ratios). The mean temperature was positively correlated with the degree of urbanization (*N* = 20, *r* = 0.641, *P* = 0.002), as was edge density (*N* = 20, *r* = 0.491, *P* = 0.028). Overall, urban areas (lower proportion of green to impervious areas) are hotter and more fragmented in terms of vegetated areas. Due to these correlations, the proportion between green and impervious surfaces has been excluded from the statistical analysis.

### Extraction of nucleic acids

Nucleic acid extraction was performed according to current laboratory protocols (Tiritelli *et al.*, 2024). Before extraction, all samples were washed in 95% ethanol to remove any potential external microbiological contaminants. Each bee was individually analyzed, placing it in a 2 mL microtube containing 800 *µ*L of DNA/RNA Shield (Zymo Research, Irvine, CA, USA) and then crushed for 3 min at 30 Hz using a TissueLyser II (Qiagen, Hilden, Germany) (Cilia *et al.*, 2022). The obtained suspensions were divided into two aliquots for separate DNA and RNA extractions. The DNA and RNA were extracted using the Quick DNA Microprep Plus Kit and the Quick RNA Microprep Plus Kit (Zymo Research), respectively, following the manufacturer's modified instructions for solid tissue processing (Mazzei *et al.*, 2019; Nanetti *et al.*, 2021b). The extracted nucleic acids were eluted in 200 *µ*L of DNAase‐RNase‐free water and stored at −80°C until qPCR analysis.

### Quantitative real‐time PCR (qPCR) assays

A quantitative Real‐Time PCR (qPCR) analysis was conducted to assess the abundance of each pathogen in the samples using the extracted DNA and RNA. DNA was used to detect the main parasites and pathogens occurring in managed bees and infecting wild bees: the fungus *Ascosphaera apis* (Onygenales; Ascosphaeraceae), the microsporidium *N. ceranae* (Microsporidia: Nosematidae), and the protozoans *A. bombi*, *L. passim*, *Crithidia mellificae* and *C. bombi* (Kinetoplastida: Trypanosomatidae). RNA was used to investigate DWV, BQCV, CBPV plus acute bee paralysis virus (ABPV), Kashmir bee virus (KBV), and sacbrood virus (SBV). The primers used for these qPCRs are listed in Table S1.

Each qPCR reaction was carried out in a total volume of 10 *µ*L, using SYBR™ green assays with forward and reverse primers and nucleic acid extract, including 2 *µ*L of extracted DNA or RNA, as described in previous studies (Cilia *et al.*, 2021, 2022). The SYBR™ PowerUp™ SYBR™ Green Master Mix (ThermoFisher, Waltham, MA, USA) and the One‐Step Power SYBR™ Green Cells‐to‐CT™ Kit (ThermoFisher Scientific) were utilized for DNA and RNA, respectively. The qPCR reactions were performed on a QuantStudio™ 3 Real‐Time PCR System (ThermoFisher Scientific), following the specific protocols for each gene sequence (James & Skinner, 2005; Chantawannakul *et al.*, 2006; Cilia *et al.*, 2018; Mazzei *et al.*, 2018; Mullins *et al.*, 2020; Buendía‐Abad *et al.*, 2023). An upper cycle threshold (Ct) of 35 was applied for positive pathogens reactions to minimize the risk of false positives.

Positive controls consisted of DNA and cDNA previously extracted from honeybees known to be infected with each pathogen, while sterile water served as a negative control. All analyses were conducted in duplicate. For each target gene, a standard curve was generated by amplifying serially diluted recombinant plasmids containing the pathogen‐specific DNA and RNA fragments, ranging from 1 × 10¹ to 1 × 10⁹ copies, in a qPCR assay on the QuantStudio™ 3 Real‐Time PCR System (ThermoFisher Scientific). This was done in accordance with previously established amplification and quantification protocols (e.g., Cilia *et al.*, 2018; Mazzei *et al.*, 2018; Buendía‐Abad *et al.*, 2023).

### Viral strand‐specific RT‐PCR

The active replication of viruses was evaluated using strand‐specific RT‐PCRs using specific primers, as previously described (Mazzei *et al.*, 2018; Nanetti *et al.*, 2021b). Positive and negative strands previously obtained from positive honeybees were used as positive controls. PCR amplified the obtained cDNAs for the viral targets, and the amplicons were visualized on a 2% agarose gel. Subsequently, the amplicons were sequenced by BMRGenomics (Padua, Italy) and analyzed using BLAST (Altschul *et al.*, 1990). All the sequences were deposited in GenBank.

### Statistical analysis

All the analyses were performed in R Statistical Software (*v. 4.3.2* R Core Team, 2023). The prevalence of pathogens (i.e., the proportion of infected individuals out of the total sampled individuals for each sampling site) was calculated along with the log‐10 transformed abundance (number of copies) of each pathogen. Abundance data were also transformed into presence (1)/absence (0) data. Spearman's correlation was used to test the correlation of the most abundant pathogens, and the results were plotted using the package *corrplot* (Wei *et al.*, 2017).

In the package *vegan* (Oksanen *et al.*, 2022), redundancy analysis (RDA) using Euclidean distance was used to test for differences in pathogen abundances among the four species. RDA was conducted on standardized abundances (mean = 0, standard deviation = 1). Homogeneity of the variance between species and sampling sites was visually checked.

For each species, pathogens with a prevalence <20% were excluded from the following analyses as they were considered too rare. First, (i) generalized linear mixed models with a binomial distribution were used to test the presence (occurrence, 0‒1) of pathogens. Then, (ii) zero‐inflated linear mixed models were used to test the log‐transformed abundance of pathogens, for each species, with a prevalence >60% (to respect the assumptions of homoscedasticity and normality). In these two models, mean temperature, edge density and sampling date (all centered and scaled) were used as fixed effects, while sampling site was included as a random effect. Finally, (iii) simple linear regressions, with temperature and edge density as predictors, were used to test the mean prevalence of pathogens (proportion of infected individuals out of all sampled individuals for each site). Mixed models were build using the packages *glmmTMB* (Brooks *et al.*, 2017) along with the package *performance* (Lüdecke *et al.*, 2021) to check statistical assumptions (VIF < 3 and the homogeneity of variance with the Bartlett test).

Plots were produced with *ggplot2* (Wickham, 2016), along with a colorblind‐friendly *viridis* palette (Garnier *et al.*, 2024). All the data are available in the Excel file (Supplementary File Dataset) in the Supporting Information. In the following sections, mean values are expressed ± standard error.

## Results

### Pathogen profile of the target species

Ten pathogen taxa were detected, while *Ascosphaera apis* and *Crithidia mellificae* were not found, although the positive controls were amplified.

**Fig. 2:**
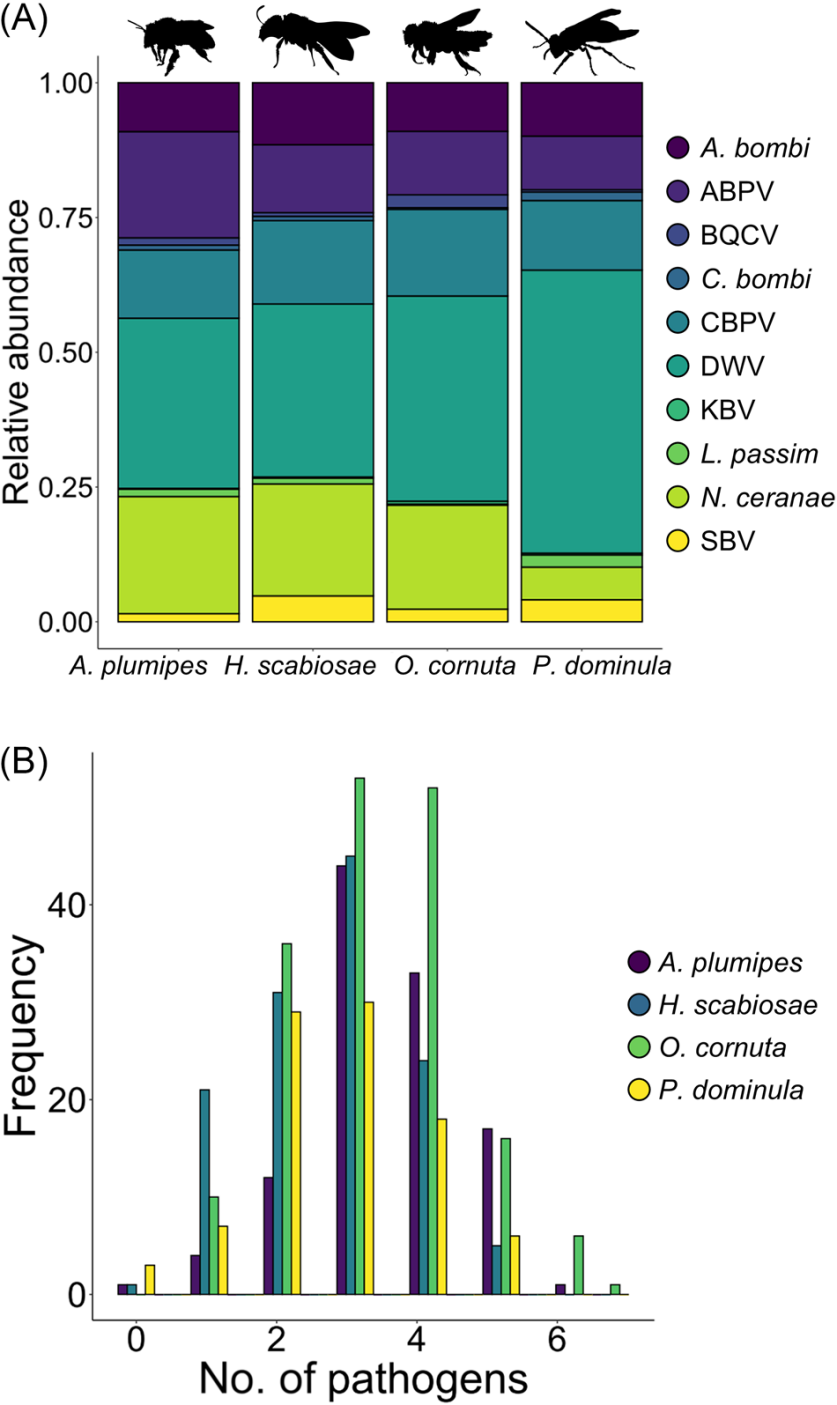
(A) Stacked barplot showing the relative abundance (0‒1) of the 10 pathogens tested in the four target species. (B) Histogram showing the frequency (i.e., number of bees/wasps) of the number of different pathogens found in each sample of the four target species (i.e., increasing coinfection from left to right). ABPV, acute bee paralysis virus; BQCV, black queen cell virus; CBPV, chronic bee paralysis virus; DWV, deformed wing virus; KBV, Kashmir bee virus; SBV, sacbrood virus.

**Fig. 3:**
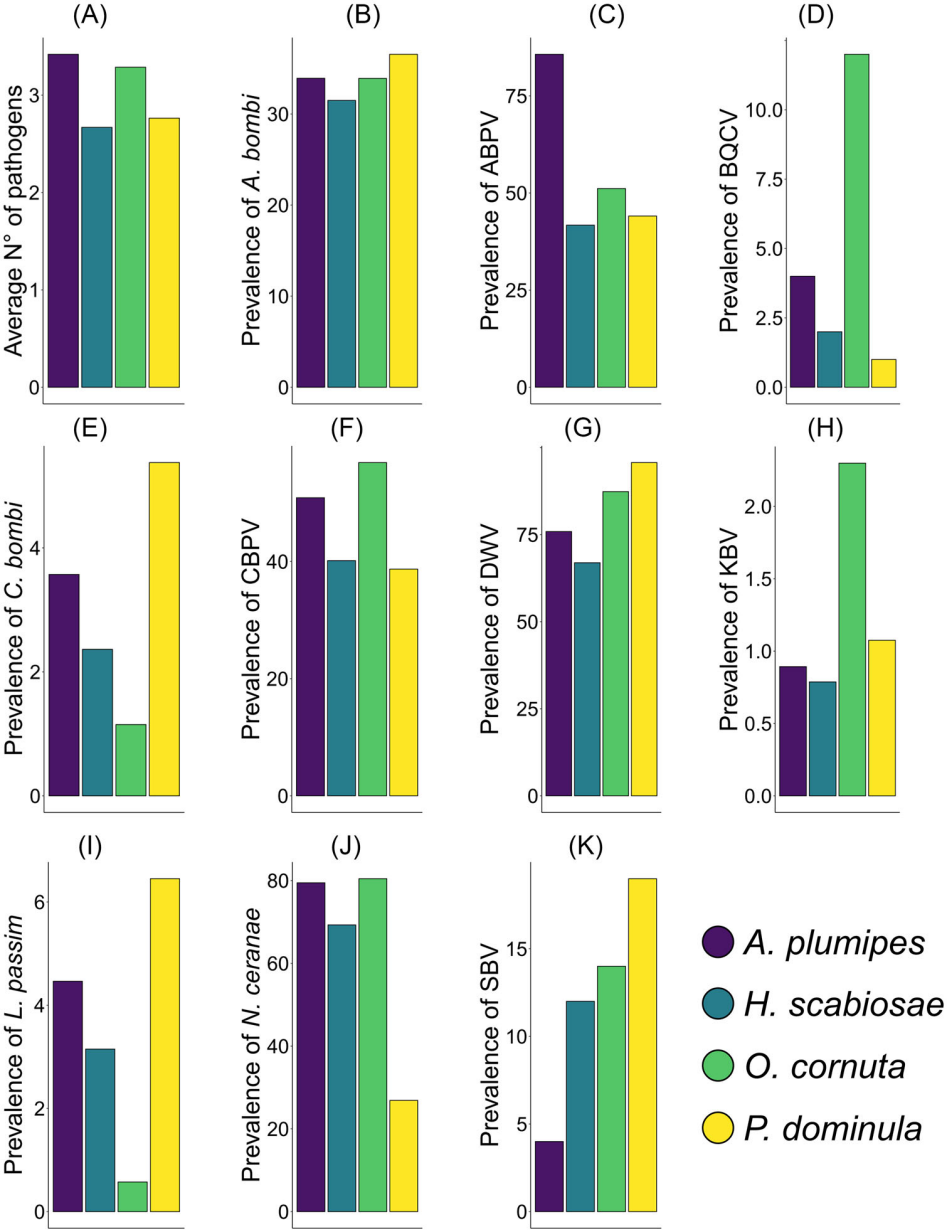
Barplot showing the average number of pathogens (A) and the average percentage (*y*‐axis = % of infected individuals out of the total tested, i.e. prevalence) of the 10 pathogens found in the four target species (B‒K). Viruses are abbreviated as follows: ABPV, acute bee paralysis virus; BQCV, black queen cell virus; CBPV, chronic bee paralysis virus; DWV, deformed wing virus; KBV, Kashmir bee virus; SBV, sacbrood virus.

The two most abundant pathogens across the four species were DWV (4.881 ± 0.123 ×10^5^ copies) and *N. ceranae* (3.362 ± 0.088 ×10^5^ copies), while the least abundant pathogens were *C. bombi* (0.097 ± 0.026 ×10^5^ copies) and KBV (0.044 ± 0.017 ×10^5^ copies), thus spanning two orders of magnitude (Figs. 2A and 3). Only five individuals were negative for all the tested pathogens (1 *A. plumipes* and *H. scabiosae*, 3 *P. dominula*), while the highest number of pathogens detected was 7 (1 *O. cornuta*) (Fig. 2B). Most *A. plumipes* and *O. cornuta* were positive for 3 or 4 pathogens. Most of *H. scabiosae* and *P. dominula* were positive for 2 or 3 pathogens (Fig. 2B). The overall pathogen profile (considering abundances) was significantly different among species (RDA, *F* = 8.080, *P* < 0.001, df = 3) and sampling sites (RDA, *F* = 2.088, *P* < 0.001, df = 19), showing an overall heterogeneity (Figs. 3 and S1).

The correlation among the abundances of the most prevalent pathogens was very low (Fig. S2). In *A. plumipes* the abundance of CBPV was negatively correlated with that of *A. bombi* (*t* = −2.047, *P* = 0.043, df = 110). In *O. cornuta* the abundance of CBPV was positively correlated with that of *N. ceranae* (*t* = 2.777, *P* = 0.006, df = 172) and *A. bombi* (*t* = 2.407, *P* = 0.017, df = 172). In *H. scabiosae* the abundance of CBPV was negatively correlated with that of DWV (*t* = −2.537, *P* = 0.012, df = 125) and the abundance of *N. ceranae* was positively correlated with that of *A. bombi* (*t* = 2.040, *P* = 0.043, df = 125). In *P. dominula* no statistically significant correlations were found.

### Effect of landscape on pathogen load

Pathogen richness (the number of pathogens detected) was never significantly dependent on temperature, edge density or sampling date (Table 1)

**Table 1:** Summary statistics of binomial generalized linear mixed models used to test the effect of temperature, green fragmentation (edge density), and sampling date on the presence/absence of the pathogens with a prevalence of >20% in each species

Species	Tested variable	Predictors	Estimate	SE	*Z*	*P*
*A. plumipes*	No. of detected pathogens	Temperature	0.090	0.063	1.423	0.155
		Edge density	0.003	0.058	0.051	0.959
		Sampling date	0.077	0.128	0.601	0.548
	*A. bombi*	Temperature	−0.136	0.238	−0.572	0.567
		**Edge density**	**0.565**	**0.250**	**2.261**	**0.024**
		Sampling date	0.276	0.504	0.547	0.585
	ABPV	Temperature	0.490	0.325	1.507	0.132
		**Edge density**	−**0.774**	**0.385**	−**2.008**	**0.045**
		Sampling date	−0.265	0.673	−0.394	0.694
	DWV	Temperature	−0.049	0.744	−0.066	0.948
		Edge density	−1.023	0.667	−1.535	0.125
		Sampling date	−0.193	1.130	−0.171	0.864
	CBPV	Temperature	0.759	0.446	1.702	0.089
		Edge density	0.146	0.404	0.362	0.717
		Sampling date	1.027	0.700	1.468	0.142
	*N. ceranae*	Temperature	0.163	0.401	0.407	0.684
		Edge density	0.357	0.384	0.930	0.353
		Sampling date	0.397	0.774	0.513	0.608
*H. scabiosae*	No. of detected pathogens	Temperature	0.066	0.058	1.126	0.260
		Edge density	0.077	0.060	1.277	0.201
		Sampling date	−0.110	0.103	−1.064	0.287
	*A. bombi*	Temperature	0.239	0.211	1.131	0.258
		Edge density	0.094	0.214	0.439	0.660
		Sampling date	−0.150	0.361	−0.415	0.678
	ABPV	Temperature	−0.010	0.334	−0.031	0.975
		Edge density	0.369	0.374	0.986	0.324
		Sampling date	0.070	0.674	0.104	0.917
	DWV	**Temperature**	**0.730**	**0.275**	**2.649**	**0.008**
		Edge density	0.159	0.296	0.536	0.592
		Sampling date	1.182	0.520	2.273	0.023
	CBPV	Temperature	0.113	0.442	0.256	0.798
		Edge density	−0.322	0.503	−0.640	0.522
		Sampling date	−1.159	0.832	−1.393	0.164
	*N. ceranae*	Temperature	0.145	0.188	0.773	0.439
		Edge density	0.169	0.218	0.774	0.439
		**Sampling date**	−**0.914**	**0.403**	−**2.271**	**0.023**
*O. cornuta*	No. of detected pathogens	Temperature	0.009	0.115	0.082	0.934
		Edge density	−0.005	0.104	−0.050	0.960
		Sampling date	0.039	0.210	0.184	0.854
	*A. bombi*	Temperature	0.208	0.301	0.690	0.490
		**Edge density**	**0.597**	**0.292**	**2.042**	**0.041**
		Sampling date	−0.104	0.529	−0.197	0.844
	ABPV	Temperature	0.246	0.345	0.713	0.476
		Edge density	−0.069	0.300	−0.229	0.819
		Sampling date	−0.485	0.645	−0.752	0.452
	DWV	Temperature	−1.897	1.736	−1.093	0.275
		Edge density	0.284	1.702	0.167	0.868
		Sampling date	−1.121	4.951	−0.226	0.821
	CBPV	Temperature	0.387	0.396	0.976	0.329
		Edge density	−0.194	0.352	−0.551	0.582
		Sampling date	0.025	0.684	0.036	0.971
	*N. ceranae*	Temperature	−0.150	0.568	−0.265	0.791
		Edge density	−0.732	0.610	−1.200	0.230
		Sampling date	−1.847	1.016	−1.818	0.069
*P. dominula*	No. of detected pathogens	Temperature	0.096	0.068	1.427	0.153
		Edge density	0.020	0.066	0.306	0.759
		Sampling date	0.101	0.112	0.897	0.370
	*A. bombi*	Temperature	0.486	0.330	1.472	0.141
		Edge density	0.554	0.312	1.777	0.076
		**Sampling date**	**1.005**	**0.495**	**2.029**	**0.042**
	ABPV	Temperature	0.188	0.279	0.672	0.501
		Edge density	0.009	0.293	0.032	0.975
		Sampling date	0.861	0.491	1.753	0.080
	DWV	Temperature	0.650	1.698	0.383	0.702
		Edge density	−1.919	1.182	−1.623	0.105
		Sampling date	1.682	1.176	1.431	0.153
	CBPV	**Temperature**	**0.680**	**0.277**	**2.458**	**0.014**
		Edge density	0.093	0.236	0.396	0.692
		Sampling date	0.384	0.389	0.988	0.323
	*N. ceranae*	Temperature	−0.121	0.281	−0.430	0.667
		Edge density	−0.114	0.325	−0.351	0.725
		Sampling date	−0.321	0.489	−0.656	0.512
	SBV	Temperature	0.355	0.390	0.910	0.363
		Edge density	0.320	0.315	1.015	0.310
		**Sampling date**	−**0.957**	**0.457**	−**2.092**	**0.036**

SE, standard error; ABPV, acute bee paralysis virus; CBPV, chronic bee paralysis virus; DWV, deformed wing virus; SBV, sacbrood virus.

*Note*: Statistically significant results (*P* < 0.05) are boldfaced.

**Fig. 4:**
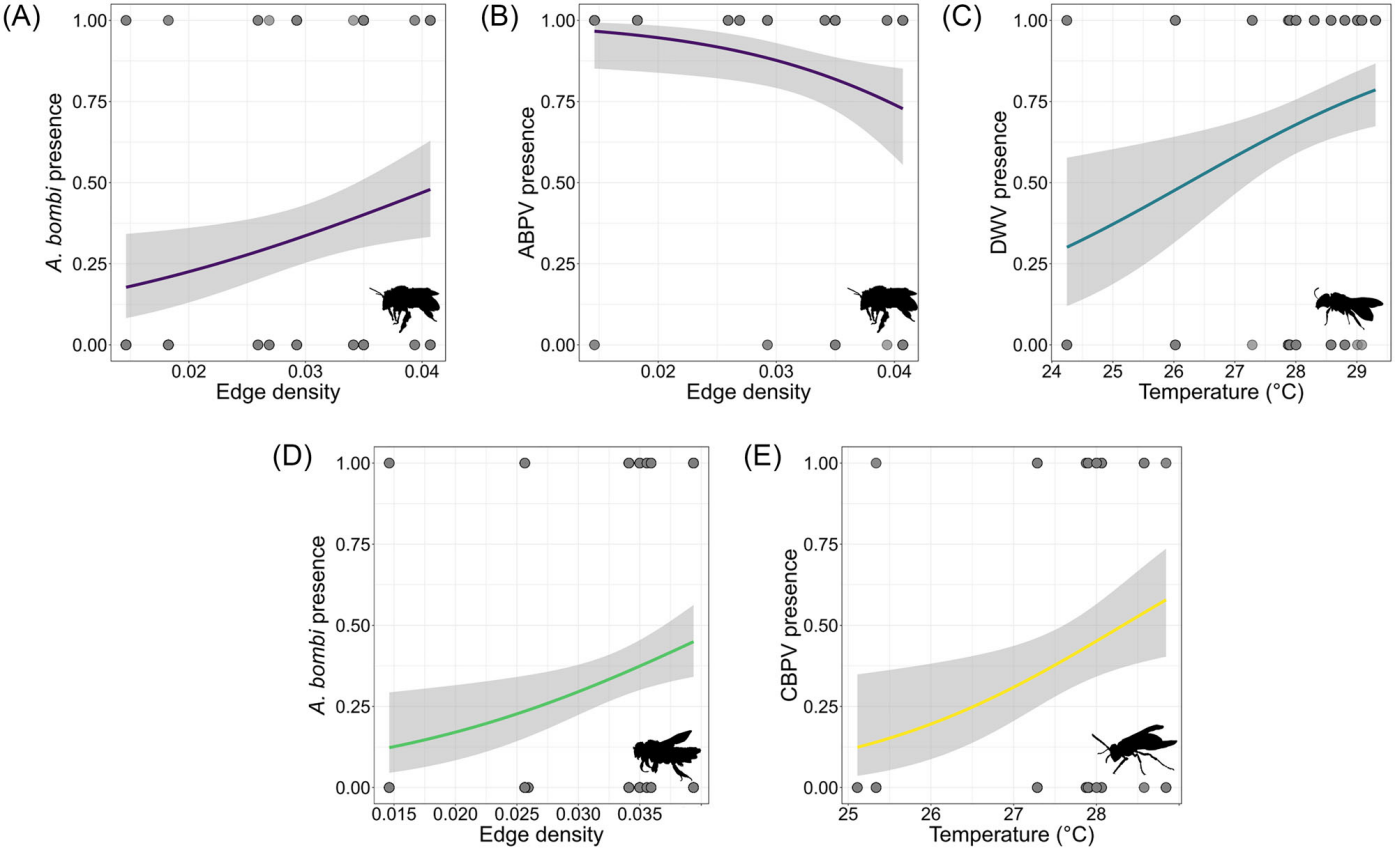
Scatterplots of statistically significant binomial linear mixed models (*P* < 0.05) used to test the presence (0‒1) of pathogens in response to temperature and green fragmentation (edge density). The line (in different colors depending on the species) and 95% confidence interval (gray) are plotted. ABPV, acute bee paralysis virus; CBPV, chronic bee paralysis virus; DWV, deformed wing virus. (A, B) *A. plumipes*, (C) *H. scabiosae*, (D) *O. cornuta*, (E) *P. dominula*.

In *A. plumipes* the presence (abundances transformed to 0‒1) of *A. bombi* increased in more fragmented sites (Fig. 4A) and the presence of ABPV decreased in more fragmented sites (Fig. 4B). In *H. scabiosae* the presence of DWV increased in warmer sites (Fig. 4C). The presence of *A. bombi* increased in more fragmented sites also in *O. cornuta* (Fig. 4D). In *P. dominula* the presence of CBPV increased in hotter sites (Fig. 4E) (Table 1). The sampling date decreased the presence of *N. ceranae* in *H. scabiosae*, and in *P. dominula* it decreased the presence of SBV and increased the presence of *A. bombi* (Table 1).

**Fig. 5:**
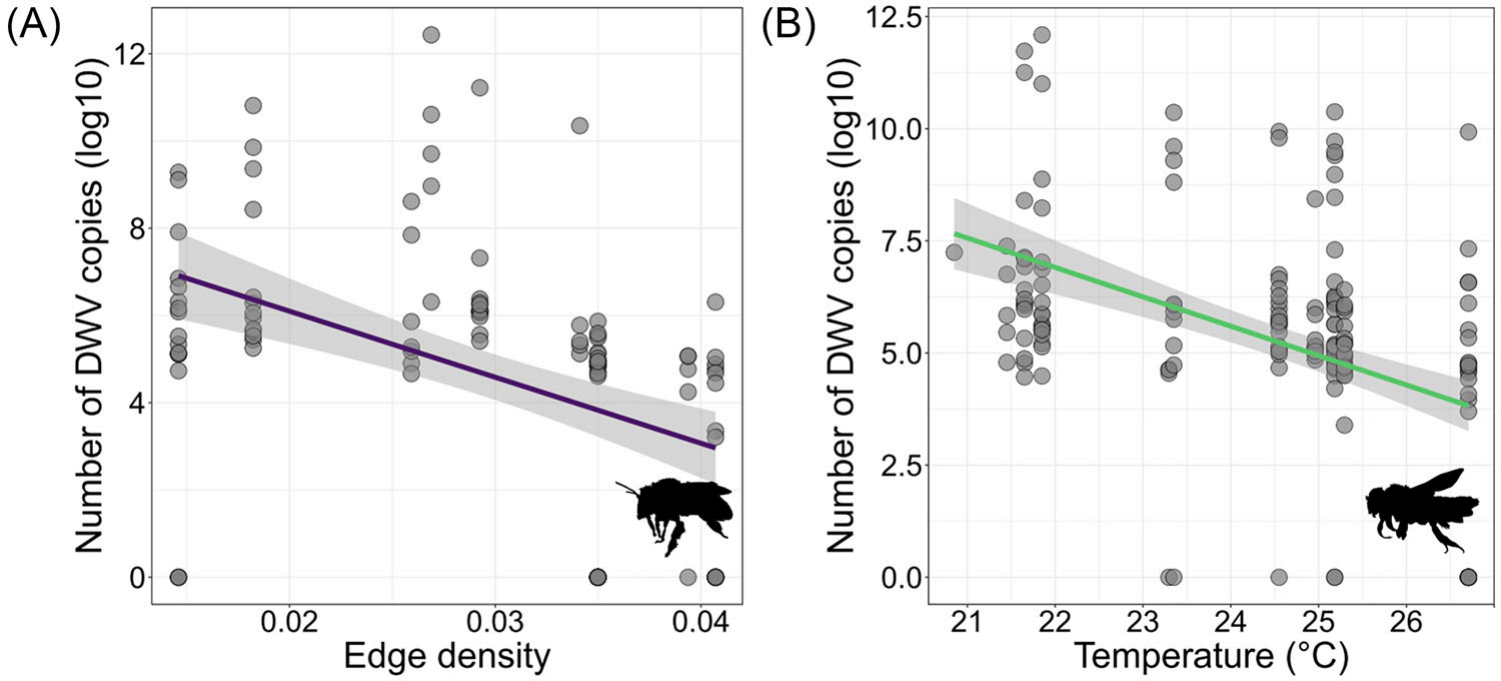
Scatterplots of statistically significant zero‐inflated linear mixed models (*P* < 0.05) the abundance (log‐transformed) of pathogens in response to temperature and green fragmentation (edge density). The line (in different colors depending on the species) and 95% confidence interval (gray) are plotted. ABPV, acute bee paralysis virus; CBPV, chronic bee paralysis virus; DWV, deformed wing virus. (A) *A. plumipes*, (B) *O. cornuta*.

The abundance (number of copies) of DWV decreased in more fragmented sites for *A. plumipes* (Fig. 5A) and in warmer sites for *O. cornuta* (Fig. 5B) (Table 2). The sampling date only significantly decreased the abundance of ABPV in *A. plumipes* (Table 2).

**Table 2:** Summary statistics of zero‐inflated linear mixed models used to test the effect of temperature, green fragmentation (edge density), and sampling date on the abundance of the pathogens with a prevalence of >60% in each species

Species	Tested variable	Predictors	Estimate	SE	*Z*	*P*
*A. plumipes*	ABPV	Temperature	0.013	0.195	0.069	0.945
		Edge density	0.123	0.180	0.685	0.494
		**Sampling date**	−**0.950**	**0.352**	−**2.702**	**0.007**
	DWV	Temperature	−0.005	0.474	−0.012	0.991
		**Edge density**	−**0.847**	**0.426**	−**1.986**	**0.047**
		Sampling date	−0.250	0.663	−0.377	0.706
	*N. ceranae*	Temperature	0.715	0.365	1.959	0.051
		Edge density	0.153	0.324	0.474	0.636
		Sampling date	−0.534	0.541	−0.986	0.324
*H. scabiosae*	DWV	Temperature	0.037	0.134	0.277	0.782
		Edge density	−0.010	0.141	−0.070	0.944
		Sampling date	0.169	0.245	0.690	0.490
	*N. ceranae*	Temperature	−0.063	0.169	−0.369	0.712
		Edge density	0.180	0.178	1.014	0.311
		Sampling date	0.100	0.290	0.346	0.730
*O. cornuta*	DWV	Temperature	−0.209	0.254	−0.824	0.410
		Edge density	−0.114	0.203	−0.564	0.573
		Sampling date	−0.822	0.426	−1.929	0.054
	*N. ceranae*	Temperature	−0.186	0.286	−0.652	0.515
		Edge density	−0.341	0.227	−1.504	0.133
		Sampling date	−0.244	0.416	−0.587	0.557
*P. dominula*	DWV	Temperature	0.148	0.250	0.593	0.553
		Edge density	−0.134	0.283	−0.475	0.635
		Sampling date	−0.199	0.407	−0.489	0.625

SE, standard error; ABPV, acute bee paralysis virus; DWV, deformed wing virus.

*Note*: Statistically significant results (*P* < 0.05) are boldfaced.

The prevalence (the proportion of positive individuals out of the total tested) was never affected neither by temperature nor edge density.

Finally, viral replication was tested in a randomized subset of 112 samples that previously scored positive to qPCR (20/112 *A. plumipes*, 25/127 *H. scabiosae*, 48/174 *O. cornuta*, and 19/93 *P. dominula*). Sequences were divided into DWV (*n* = 49), ABPV (*n* = 28), CBPV (*n* = 27), KBV (*n* = 7), BQCV (*n* = 9), and SBV (*n* = 11) accordingly to their relative presence across the samples. All these virus samples resulted replicative. The sequences were deposited in GenBank: DWV Accession Number (PQ295940‐PQ295988), CBPV (PQ295989‐PQ296015), KBV (PQ296036‐PQ296042), SBV (PQ296025‐PQ296035), ABPV (PQ295912‐PQ295939), and BQCV (PQ296016‐PQ296024).

## Discussion

The results show the intraspecific occurrence of pathogens in four, still poorly studied, Hymenoptera species exposed to different urban pressures (e.g., Tommasi *et al.*, 2023; Cuvillier‐Hot *et al.*, 2024), but without providing information on pathogen transmission (Deutsch *et al.*, 2023).

It was shown how consistent the presence of pathogens is in wild Hymenoptera. The most common pathogens were the viruses DWV and ABPV and the microsporidian *N. ceranae* confirming previous findings (Cilia *et al.*, 2022). Pathogens found with over 10^6^ copies generally produce symptomatic infection in honeybees (Martín‐Hernández *et al.*, 2011; Mazzei *et al.*, 2014; Cilia *et al.*, 2020), and this threshold was consistently found in this study. Whether this threshold applies to other wild insects remains to be tested and was beyond this study's scope. Nevertheless, there are cases of deformed wings in *Bombus*, *Megachile*, *Xylocopa* (bees), and the wasp *Vespa* (Genersch *et al.*, 2006; Lucia *et al.*, 2014; Cilia *et al.*, 2021, 2024; Flaminio *et al.*, 2023; Lanner *et al.*, 2023). Conversely, there is less clarity regarding the effects of *N. ceranae* in wild bees (Müller *et al.*, 2019; Eskov *et al.*, 2022), since ingested spores can pass ungerminated through the intestinal tract and therefore being not harmful (Gisder *et al.*, 2020).

The still poorly studied *A. bombi* had a relatively high prevalence across the four target species, partially agreeing with a previous study carried out in Milan where *Bombus terrestris* was found infected by *A. bombi* (Tommasi *et al.*, 2023). *Apcystis bombi* appears to be remarkable among neogregarines in having a wide range of host species other than its primary bumblebee hosts, including most of the pollinating Hymenoptera (Plischuk & Lange, 2024). Furthermore, the pathological effects of *A. bombi* are largely unknown with suggestions of chronic effects (Lange & Lord, 2012), high mortality in hibernating *Bombus* queens (Rutrecht & Brown, 2008), or higher sucrose sensitivity and a lower lipid: body size ratio in bumblebees (Graystock *et al.*, 2016).

Interestingly, a high presence of the viruses DWV, CBPV, ABPV, and the neogregarine *A. bombi* was also found in the wasp *P. dominula*. Bee pathogens are mostly known in wasp predators of *A. mellifera* (e.g., *Vespa crabro* Linnaeus, 1761, *V. velutina* Lepeletier, 1836, *V. orientalis* Linnaeus, 1771) (Forzan *et al.*, 2017; Mazzei *et al.*, 2019; Cilia *et al.*, 2024; Power *et al.*, 2024), while less is known in *Polistes* (caterpillar hunters). The occurrence of pathogens may be due to the shared landscape elements, bypassing direct bee‐wasp contact (Schmid‐Hempel & Cremer, 2021). Furthermore, these results add to the still scarce evidence on the presence of the protozoan *L. passim* and the virus KBV in wild populations (Cilia *et al.*, 2023). In addition, the presence of CBPV of this study is relevant since there has been evidence of a widespread nationwide increase of this virus in the last years (Zavatta *et al.*, 2024). Taken together, the presence of many pathogens is important evidence that a phenomenon of pathogen transfers is occurring within urban insect populations.

Our two nonmutually exclusive hypotheses, that is, (i) higher temperatures reduce pathogen presence/abundance (UHI hypothesis), and (ii) higher greenspace fragmentation increases pathogen presence/abundance (AE hypothesis) were partially supported. In *H. scabiosae* and *P. dominula* mean temperature (UHI effect) was positively correlated with the occurrence of DWV and CBPV respectively. This does not agree with the UHI hypothesis, but it is supported in the literature with studies on honeybees and bumblebees (Youngsteadt *et al.*, 2015). This may suggest that urban habitats (sites with the highest mean temperatures) offer greater opportunities for parasite transmission (Goulson *et al.*, 2012). However, it has also been shown that urbanization increased the expression of genes linked to immunometabolism, supposedly in response to the stressful conditions of cities. The induction of these genes is likely at the root of any immune activation (Cuvillier‐Hot *et al.*, 2024) that may counteract the possible increase in pathogen infection in cities.

Instead, the “amplification effect” (AE) hypothesis was more supported. More fragmented areas were associated with higher occurrences of the protozoan *A. bombi* in the bees *A. plumipes* and *O. cornuta*. A higher density of insects in these fragmented parks may increase the likelihood of pathogen transmission (Durrer & Schmid‐Hempel, 1994). However, it was also found that ABPV presence in *A. plumipes* decreased in more fragmented sites. This is in accordance with a previous study in the same area (Tommasi *et al.*, 2023). Therefore, other effects of the environment, such as the presence and abundance of honeybees in the sampled locations cannot be excluded.

The results about the abundances of pathogens supported the UHI hypothesis. In *O. cornuta*, higher temperatures (urban areas) were associated with lower number of DWV copies. Laboratory studies on honeybees (Dalmon *et al.*, 2019; Palmer‐Young *et al.*, 2023; Abou‐Shaara *et al.*, 2024) showed a negative effect of temperature on pathogens, especially viruses, while field‐collected wild bees presented lower pathogen load at higher ambient temperatures (Piot *et al.*, 2022). In addition, the expression of antimicrobial peptide genes was found to increase in response to increasing temperature in two *Apis* species (Li *et al.*, 2022). Therefore, a reduction in viral load may be due to direct thermal inhibition, although alternative mechanisms cannot be excluded, such as the induction of antimicrobial peptide genes (Xu & James, 2012). However, this was not the case for the abundance of DWV in *A. plumipes*. Albeit being somehow coherent with the results from *O. cornuta*—lower abundance in more urbanized areas—this result disagrees with the AE hypothesis and with what was found in honeybees where urbanization increased pathogen pressure (Youngsteadt *et al.*, 2015). In addition, in accordance with previous findings (Olgun *et al.*, 2020), the prevalence (i.e., number of infected individuals out of the total sampled in a site) was not affected by the urban landscape.

Finally, the observed replication of all viruses, consistent with other studies (Cilia *et al.*, 2022), may suggests that the target species do not act as simple carriers of pathogens. Although no conclusions can be drawn about transmission routes, monitoring infections in wild populations is likely to provide a better understanding of transmission (Piot *et al.*, 2022).

Despite some nonnegligible shortcomings, it has been shown that there is a consistent presence of pathogens in the four target hymenopteran species, and that urbanization, tested in terms of temperature and greenspace fragmentation, plays a role in modulating pathogen presence and to a lesser extent abundance. Urban areas are likely to increase the presence of pathogens, while decreasing the abundance of DWV. These results can have conservation implications as they provide some of the first evidence of the effects of land‐use change on pathogens of wild bees and wasps. More cohesive green spaces and the reduction of anthropic pressures would likely reduce the pathogen load on wild bees and wasps. Future studies may also consider testing whether the structure of the Hymenoptera community in different urban settings influences the incidence of generalist parasites.

## Author contributions

AF: Data curation, Formal analysis, Investigation, Methodology, Visualization, Writing—original draft, Writing—review & editing. GC: Conceptualization, Data curation, Formal analysis, Investigation, Methodology, Resources, Writing—original draft, Writing—review & editing. CP: Conceptualization, Investigation, Formal analysis, Funding acquisition, Project administration, Resources, Supervision, Validation, Writing—original draft, Writing—review & editing.

## Funding

Project funded under the National Recovery and Resilience Plan (NRRP), Mission 4 Component 2 Investment 1.4 ‐ Call for tender No. 3138 of December 16, 2021, rectified by Decree n.3175 of December 18, 2021 of Italian Ministry of University and Research funded by the European Union—NextGenerationEU, Project code CN_00000033, Concession Decree No. 1034 of June 17, 2022 adopted by the Italian Ministry of University and Research, CUP, H43C22000530001 Project title “National Biodiversity Future Center— NBFC.”

## Disclosure

The authors declare that they have no known competing financial interests or personal relationships that could appear to have influenced the work reported in this paper.

## Supporting information




**Supplementary File Dataset** Data used for statistical analysis.

**Fig. S1** Representation of the Redundancy Analysis (RDA) testing the difference in the pathogen profile across the four target species. ***P < 0.001, in parenthesis proportion of variance explained.
**Fig. S2** Correlation matrices testing the co‐occurrence of the most abundant pathogens in the four target species. The scale bar at the bottom represents Spearman's correlation (−1 in red to 1 in blue). (A) *A. plumipes*, (B) *H. scabiosae*, (C) *O. cornuta*, (D) *P. dominula*. Viruses are abbreviated as follows: ABPV, acute bee paralysis virus; CBPV, chronic bee paralysis virus; DWV, deformed wing virus; SBV, sacbrood virus.

**Table S1**. List of primers used to detect parasites and viruses. DWV, deformed wing virus; BQCV, black queen cell virus; CBPV, chronic bee paralysis virus; ABPV, acute bee paralysis virus; KBV, Kashmir bee virus; SBV, sacbrood virus. Full references are given at the end of the document.

**Table S2**. Summary statistics of the ordinary linear models used to test the effect of urbanization on the prevalence of the pathogen in each species. SE: estimate standard error. Statistically significant results (*P* < 0.05) are boldfaced. Viruses are abbreviated as follows: ABPV, acute bee paralysis virus; CBPV, chronic bee paralysis virus; DWV, deformed wing virus; SBV, sacbrood virus.

## Data Availability

All data are available in the Excel file (Supplementary File Dataset) in the Supporting Information. Sequences were submitted to Genbank under the accession numbers found in the results section.
