# Supplementary material for: Urban landscapes tend to increase the presence of pathogenic protozoa, microsporidia and viruses, but likely decrease the abundance of viruses in wild bees and wasps
Source: Insect Sci. 2025 Jul 27;32(6):1911–25. doi: 10.1111/1744-7917.70137 (PMC12717336; doi:10.1111/1744-7917.70137)
Supplement: Supplementary file 2 — Fig. S1 Representation of the Redundancy Analysis (RDA) testing the difference in the pathogen profile across the four target species. ***P < 0.001, in parenthesis proportion of variance explained. Fig. S2 Correlation matrices testing the co‐occurrence of the most abundant pathogens in the four target species. The scale bar at the bottom represents Spearman's correlation (−1 in red to 1 in blue). (A) A. plumipes, (B) H. scabiosae, (C) O. cornuta, (D) P. dominula. Viruses are abbreviated as follows: ABPV, acute bee paralysis virus; CBPV, chronic bee paralysis virus; DWV, deformed wing virus; SBV, sacbrood virus. [file INS-32-1911-s002.pdf]

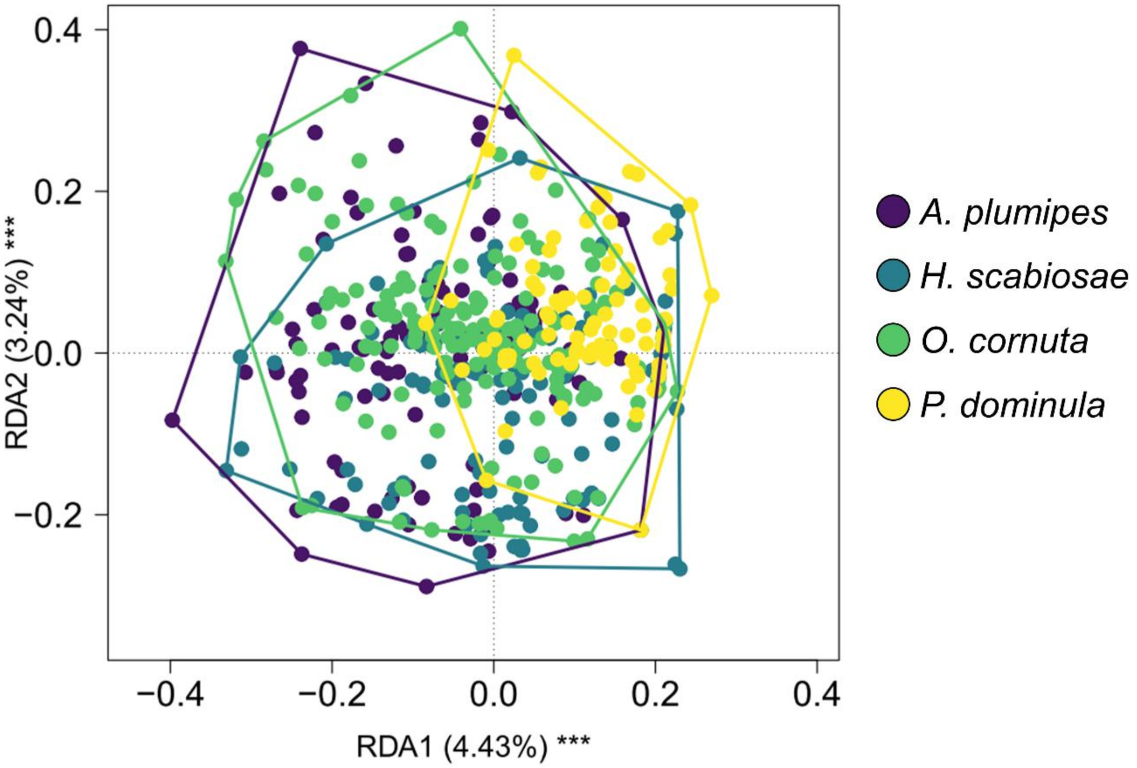

Figure S1. Representation of the Redundancy Analysis (RDA) testing the difference in the pathogen profile across the four target species. ***: $P < 0.001$, in parenthesis proportion of variance explained.

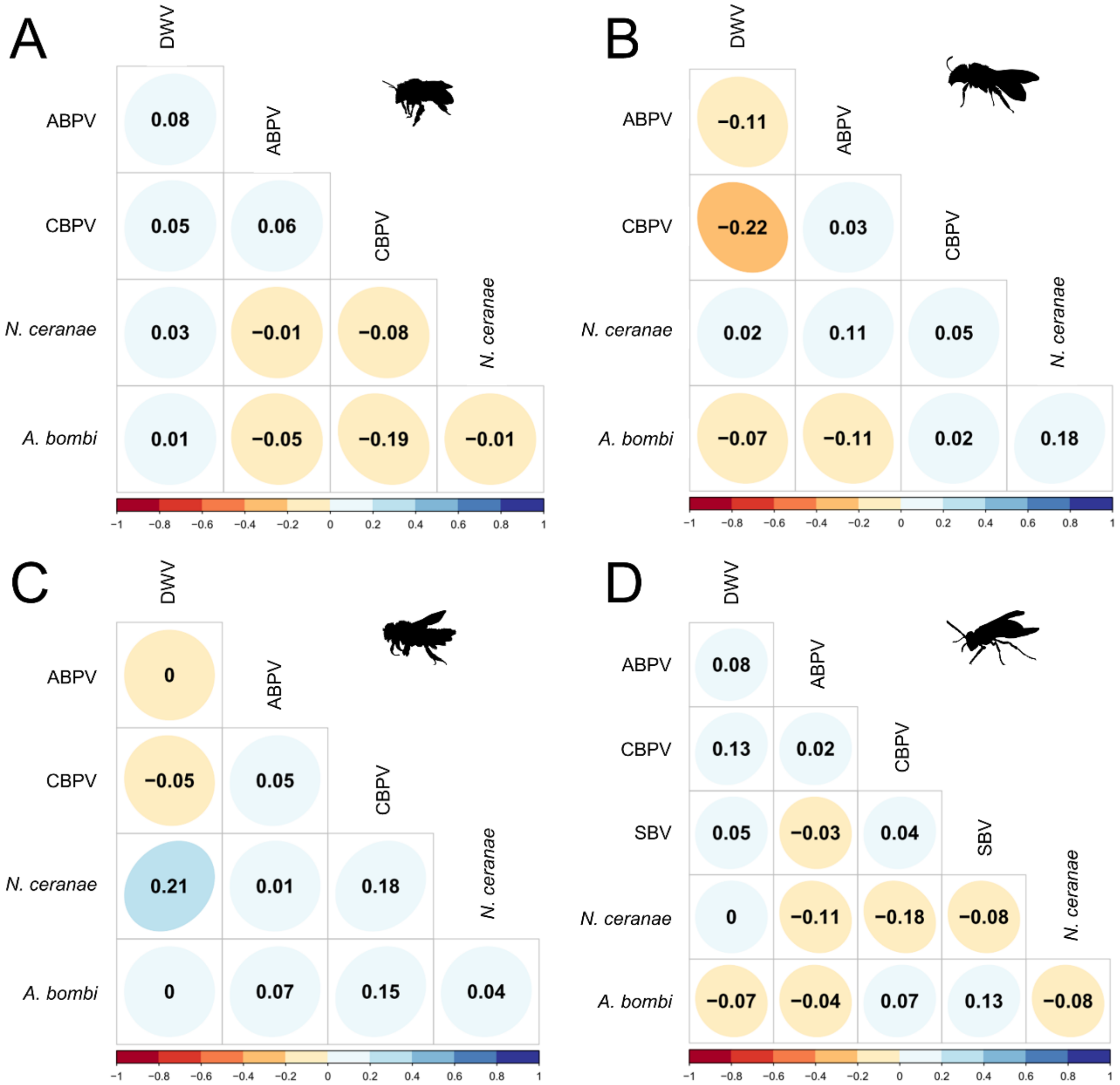

Figure S2. Correlation matrices testing the co-occurrence of the most abundant pathogens in the four target species. The scale bar at the bottom represents Spearman's correlation (-1 in red to 1 in blue). A) *A. plumipes*, B) *H. scabiosae*, C) *O. cornuta*, D) *P. dominula.* Viruses are abbreviated as follows: ABPV (acute bee paralysis virus), CBPV (chronic bee paralysis virus), DWV (deformed wing virus), SBV (sacbrood virus).
