# Supplementary material for: Urban landscapes tend to increase the presence of pathogenic protozoa, microsporidia and viruses, but likely decrease the abundance of viruses in wild bees and wasps
Source: Insect Sci. 2025 Jul 27;32(6):1911–25. doi: 10.1111/1744-7917.70137 (PMC12717336; doi:10.1111/1744-7917.70137)
Supplement: Supplementary file 3 — Table S1. List of primers used to detect parasites and viruses. DWV, deformed wing virus; BQCV, black queen cell virus; CBPV, chronic bee paralysis virus; ABPV, acute bee paralysis virus; KBV, Kashmir bee virus; SBV, sacbrood virus. Full references are given at the end of the document. [file INS-32-1911-s004.docx]

**Table S1.** List of primers used to detect parasites and viruses. DWV, deformed wing virus; BQCV, black queen cell virus; CBPV, chronic bee paralysis virus; ABPV, acute bee paralysis virus; KBV, kashmir bee virus; SBV (sacbrood virus). Full references are given at the end of the document.

| **Target** | **Primer name** | **Sequence (5’-3’)** | **Reference** |
| --- | --- | --- | --- |
| *Ascosphaera apis* | A_apis_3-F1  A_apis_3-R1 | TGTCTGTGCGGCTAGGTG  CCACTAGAAGTAAATGATGGTTAGA | James and Skinner (2005) |
| *Apicystis bombi* | Apicyst357F Apicyst357R | AGCGATGGATGTCTTGGGTC CCTAGTTAGTTTCTTTTCCTCCGC | Mullins et al. (2020) |
| *Nosema ceranae* | Hsp70_F  Hsp70_R | GGGATTACAAGTGCTTAGAGTGATT  TGTCAAGCCCATAAGCAAGTG | Cilia et al. (2018) |
| *Lotmaria passim* | TOPII_F  TOPII_R | GGCCATGGAAATACTCGAGTCT ACCTTGCCTTCCTTCTTGAGATT | Buendia-Abad et al. (2023) |
| *Crithidia mellificae* | RPB1_F  RPB2_R | TGGTGGGTGCGATTACGAA TCATTGAAGATGACGTGGATAAGC | Buendia-Abad et al. (2023) |
| *Crithidia bombi* | Cytb_F  Cytb_R | TTTTGCCATGCACTATGATGTCT AACCTATTACAGGCACAGTTGCTAAA | Buendia-Abad et al. (2023) |
| DWV | DWV Fw 8450  DWV Rev 8953 | TGGCATGCCTTGTTCACCGT  CGTGCAGCTCGATAGGATGCCA | Mazzei et al. (2018) |
| BQCV | BQCV 9195F  BQCV 8265R | GGTGCGGGAGATGATATGGA  GCCGTCTGAGATGCATGAATAC | Chantawannakul et al. (2006) |
| CBPV | CPV 304F 79  CPV 371R | TCTGGCTCTGTCTTCGCAAA  GATACCGTCGTCACCCTCATG | Chantawannakul et al. (2006) |

**References**

Buendía-Abad, M., Martín-Hernández, R., & Higes, M. (2023). Trypanosomatids in honey bee colonies in Spain: A new specific qPCR method for specific quantification of *Lotmaria passim*, *Crithidia mellificae* and *Crithidia bombi*. *Journal of Invertebrate Pathology*, 201, 108004.

Chantawannakul, P., Ward, L., Boonham, N., & Brown, M. (2006). A scientific note on the detection of honeybee viruses using real-time PCR (TaqMan) in Varroa mites collected from a Thai honeybee (*Apis mellifera*) apiary. *Journal of invertebrate pathology*, *91*(1), 69-73.

Cilia, G., Cabbri, R., Maiorana, G., Cardaio, I., Dall’Olio, R., & Nanetti, A. (2018). A novel TaqMan® assay for Nosema ceranae quantification in honey bee, based on the protein coding gene Hsp70. *European Journal of Protistology*, *63*, 44-50.

James, R. R., & Skinner, J. S. (2005). PCR diagnostic methods for Ascosphaera infections in bees. *Journal of Invertebrate Pathology*, 90(2), 98-103.

Mazzei, M., Forzan, M., Cilia, G., Sagona, S., Bortolotti, L., & Felicioli, A. (2018). First detection of replicative deformed wing virus (DWV) in Vespa Velutina Nigrithorax. *Bulletin of Insectology*, 71(2).

Mullins, J. L., Strange, J. P., & Tripodi, A. D. (2020). Why are queens broodless? Failed nest initiation not linked to parasites, mating status, or ovary development in two bumble bee species of Pyrobombus (Hymenoptera: Apidae: Bombus). *Journal of Economic Entomology*, 113(2), 575-581.
