# Supplementary material for: Urban landscapes tend to increase the presence of pathogenic protozoa, microsporidia and viruses, but likely decrease the abundance of viruses in wild bees and wasps
Source: Insect Sci. 2025 Jul 27;32(6):1911–25. doi: 10.1111/1744-7917.70137 (PMC12717336; doi:10.1111/1744-7917.70137)
Supplement: Supplementary file 4 — Table S2. Summary statistics of the ordinary linear models used to test the effect of urbanization on the prevalence of the pathogen in each species. SE: estimate standard error. Statistically significant results (P < 0.05) are boldfaced. Viruses are abbreviated as follows: ABPV, acute bee paralysis virus; CBPV, chronic bee paralysis virus; DWV, deformed wing virus; SBV, sacbrood virus. [file INS-32-1911-s001.docx]

**Table S2.** Summary statistics of the ordinary linear models used to test the effect of temperature and green fragmentation (edge density) on the prevalence of the pathogen (i.e., proportion of infected individuals out of the total sampled for each site). S.E.: estimate standard error.

Statistically significant results (P < 0.05) are boldfaced. Viruses are abbreviated as follows: ABPV (acute bee paralysis virus), CBPV (chronic bee Paralysis Virus), DWV (deformed wing virus), SBV (sacbrood virus).

| **Species** | **Tested variable** | **Predictors** | **Estimate** | **S.E.** | **T** | **P** |
| --- | --- | --- | --- | --- | --- | --- |
| *A. plumipes* | *A. bombi* | Temperature | 0.459 | 0.785 | 0.585 | 0.580 |
|  |  | Edge density | 1050.725 | 436.939 | 2.405 | 0.053 |
|  | ABPV | Temperature | 6.144 | 5.478 | 1.122 | 0.305 |
|  |  | Edge density | -8.477 | 4.592 | -1.846 | 0.114 |
|  | CBPV | Temperature | 10.645 | 13.087 | 0.813 | 0.447 |
|  |  | Edge density | -2.257 | 10.972 | -0.206 | 0.844 |
|  | DWV | Temperature | 1.281 | 9.491 | 0.135 | 0.897 |
|  |  | Edge density | -12.183 | 7.957 | -1.531 | 0.177 |
|  | *N. ceranae* | Temperature | 1.402 | 9.069 | 0.155 | 0.882 |
|  |  | Edge density | 4.495 | 7.603 | 0.591 | 0.576 |
| *H. scabiosae* | *A. bombi* | Temperature | 6.043 | 3.601 | 1.678 | 0.124 |
|  |  | Edge density | 1.472 | 4.268 | 0.345 | 0.737 |
|  | ABPV | Temperature | -0.563 | 7.257 | -0.078 | 0.940 |
|  |  | Edge density | 8.148 | 8.602 | 0.947 | 0.366 |
|  | CBPV | Temperature | 7.513 | 8.739 | 0.860 | 0.410 |
|  |  | Edge density | -5.476 | 10.358 | -0.529 | 0.609 |
|  | DWV | Temperature | 9.035 | 6.157 | 1.467 | 0.173 |
|  |  | Edge density | 5.083 | 7.298 | 0.697 | 0.502 |
|  | *N. ceranae* | Temperature | 6.869 | 4.964 | 1.384 | 0.197 |
|  |  | Edge density | 4.599 | 5.884 | 0.782 | 0.453 |
| *O. cornuta* | *A. bombi* | Temperature | 9.305 | 7.817 | 1.190 | 0.287 |
|  |  | Edge density | 10.423 | 6.745 | 1.545 | 0.183 |
|  | ABPV | Temperature | -0.760 | 11.251 | -0.068 | 0.949 |
|  |  | Edge density | -4.416 | 9.708 | -0.455 | 0.668 |
|  | CBPV | Temperature | 12.725 | 9.222 | 1.380 | 0.226 |

|  |  | Edge density | -5.307 | 7.957 | -0.667 | 0.534 |
| --- | --- | --- | --- | --- | --- | --- |
|  | DWV | Temperature | -12.470 | 9.602 | -1.299 | 0.251 |
|  |  | Edge density | -0.986 | 8.285 | -0.119 | 0.910 |
|  | *N. ceranae* | Temperature | 2.449 | 8.059 | 0.304 | 0.773 |
|  |  | Edge density | -10.675 | 6.954 | -1.535 | 0.185 |
| *P. dominula* | *A. bombi* | Temperature | 7.514 | 8.500 | 0.884 | 0.406 |
|  |  | Edge density | 7.254 | 9.800 | 0.740 | 0.483 |
|  | ABPV | Temperature | -0.797 | 9.650 | -0.083 | 0.936 |
|  |  | Edge density | -1.161 | 11.125 | -0.104 | 0.920 |
|  | CBPV | Temperature | 13.463 | 7.637 | 1.763 | 0.121 |
|  |  | Edge density | 1.460 | 8.805 | 0.166 | 0.873 |
|  | DWV | Temperature | 1.338 | 2.067 | 0.648 | 0.538 |
|  |  | Edge density | -4.638 | 2.383 | -1.947 | 0.093 |
|  | *N. ceranae* | Temperature | -1.634 | 6.970 | -0.234 | 0.821 |
|  |  | Edge density | 2.708 | 8.036 | 0.337 | 0.746 |
|  | SBV | Temperature | 7.207 | 6.202 | 1.162 | 0.283 |
|  |  | Edge density | 3.622 | 7.151 | 0.507 | 0.628 |
